# Hydroxychloroquine in lupus or rheumatoid arthritis pregnancy and risk of major congenital malformations: a population-based cohort study

**DOI:** 10.1093/rheumatology/keae168

**Published:** 2024-03-13

**Authors:** Ngoc V Nguyen, Elisabet Svenungsson, Annica Dominicus, Maria Altman, Karin Hellgren, Julia F Simard, Elizabeth V Arkema

**Affiliations:** Clinical Epidemiology Division, Department of Medicine Solna, Karolinska Institutet, Stockholm, Sweden; Division of Rheumatology, Department of Medicine Solna, Karolinska Institutet, Karolinska University Hospital, Stockholm, Sweden; Clinical Epidemiology Division, Department of Medicine Solna, Karolinska Institutet, Stockholm, Sweden; Clinical Epidemiology Division, Department of Medicine Solna, Karolinska Institutet, Stockholm, Sweden; Clinical Epidemiology Division, Department of Medicine Solna, Karolinska Institutet, Stockholm, Sweden; Clinical Epidemiology Division, Department of Medicine Solna, Karolinska Institutet, Stockholm, Sweden; Division of Immunology and Rheumatology, Department of Medicine, Stanford School of Medicine, Stanford, CA, USA; Department of Epidemiology and Population Health, Stanford School of Medicine, Stanford, CA, USA; Clinical Epidemiology Division, Department of Medicine Solna, Karolinska Institutet, Stockholm, Sweden

**Keywords:** hydroxychloroquine, systemic lupus erythematosus, rheumatoid arthritis, major congenital malformations, autoimmune disease, inverse probability of treatment weighting

## Abstract

**Objectives:**

To assess the infant risk of major congenital malformations (MCM) associated with first-trimester exposure to hydroxychloroquine (HCQ) among mothers with systemic lupus erythematosus (SLE) or rheumatoid arthritis (RA).

**Methods:**

This population-based cohort study utilized Swedish nationwide registers and included all singleton births (2006–2021) among individuals with prevalent SLE or RA in Sweden. The exposure was filling ≥1 HCQ prescription during the first trimester. The outcome was infant MCM within 1 year of birth. Inverse probability of treatment weighting was applied to adjust for potential confounders (e.g. maternal smoking, body mass index, pregestational diabetes and corticosteroids). Modified Poisson regression models with robust variance were used to estimate risk ratios (RR) and 95% CI.

**Results:**

We included 1007 births (453 exposed) and 2500 births (144 exposed) in the SLE and RA cohorts, respectively. The MCM risks in the SLE overall cohort, exposed and unexposed groups were 3.6%, 3.7% and 3.4%, respectively. The corresponding figures in the RA cohort were 4.4%, 5.6% and 4.3%, respectively. The adjusted RRs (95% CI) were 1.29 (0.65, 2.56) in the SLE cohort, 1.32 (0.56, 3.13) in the RA cohort and 1.30 (0.76, 2.23) in the pooled analysis. The adjusted risk difference (exposed *vs* unexposed) was small (0.9% in SLE and 1.3% in RA). Sensitivity analyses examining different exposure and outcome windows yielded similar findings.

**Conclusion:**

First-trimester exposure to HCQ was not associated with a significantly increased risk of MCM. HCQ’s benefits may outweigh the risks in managing SLE or RA during pregnancy.

## Graphical Abstract

**Figure:**
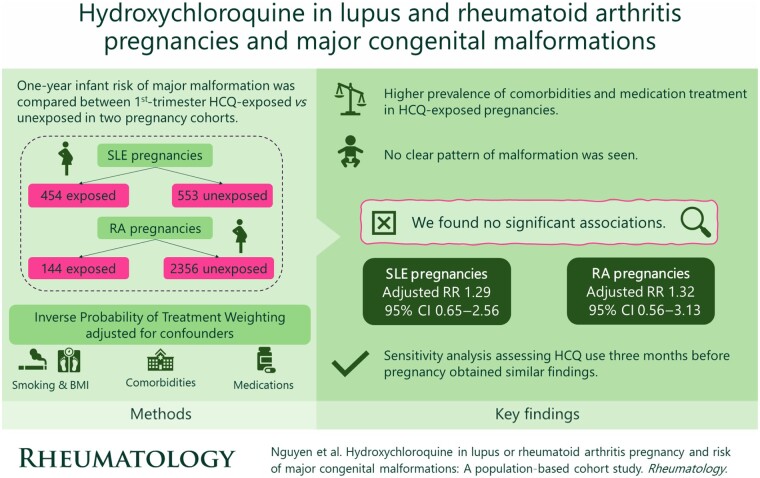

Rheumatology key messages

- The association between hydroxychloroquine and major congenital malformations was examined in SLE/RA pregnancies in Sweden.
- First-trimester hydroxychloroquine exposure was not significantly associated with malformations, with no clear malformation patterns observed.
- Results remained similar when considering hydroxychloroquine use before pregnancy and/or varying malformation assessment periods.

## Introduction

Individuals with systemic lupus erythematosus (SLE) and rheumatoid arthritis (RA) are often of childbearing age [1]. SLE or RA pregnancies carry a higher risk of adverse outcomes than pregnancies of healthy women [1–3]. Pharmacological treatment is often recommended before and during pregnancy to control disease activity and reduce the risk of adverse maternal and fetal outcomes [2, 3].

Hydroxychloroquine (HCQ), an antimalarial agent and a DMARD, is generally considered safe during pregnancy and recommended for SLE or RA pregnancies unless contraindicated [2–5]. In SLE, HCQ continuation during pregnancy has been reported to reduce flare risk [6, 7] and corticosteroid dose [8] and is not associated with increased risks of common obstetrical outcomes [9, 10]. Although HCQ has long been used for pregnant women, its teratogenicity is still questioned because it can cross the placenta [11] and interfere with DNA synthesis [12].

Most epidemiological studies examining the association between HCQ and major congenital malformations (MCM) have been underpowered (*n* < 500) and lacked focus on a particular indication of HCQ. The majority of these studies reported no statistically significant association [10, 13–18]. However, one study from the USA comparing 1867 first-trimester HCQ-exposed and 19 080 HCQ-unexposed pregnancies in mothers with any rheumatic disease revealed an increased risk of MCM (adjusted risk ratio [RR] 1.26; 95% CI: 1.04, 1.54) [19]. Following this study, the European Medicines Agency in February 2023 updated the background information of HCQ use during pregnancy [20, 21], replacing all the non-significant findings of prior research with the significant association reported in the study from the USA. Both the study from the USA and the update have generated major concerns among rheumatology societies, clinicians, patients and policymakers [22–24].

Given that HCQ is currently recommended for all pregnant women with SLE and the limited and inconsistent findings in existing literature, this study aimed to assess the risk of MCM associated with HCQ exposure during early pregnancy among women with SLE or RA.

## Methods

### Study population and design

This population-based cohort study included all singleton births resulting from pregnancies with prevalent SLE or RA from August 2006 to December 2021 in Sweden. Pregnancies and births were identified from the Swedish Medical Birth Register (MBR), which includes information on >98% of deliveries in Sweden since 1973 [25]. All live births are included, while stillbirths only from gestational week 28 (1973–2008) or from week 22 (since June 2008) are included. From the Swedish National Patient Register (NPR), which includes all inpatient hospitalizations since 1964 and outpatient specialist visits since 2001 in Sweden [26], we identified individuals with SLE or RA by requiring ≥2 International Classification of Disease (ICD)-coded visits (not on the same date) for each respective diagnosis. At least one visit had to be with a specialist that typically diagnoses or treats SLE or RA (e.g. rheumatology, internal medicine). This identification method has been used before in the NPR and has shown good accuracy [27, 28]. To identify pregnancies with prevalent SLE or RA, we linked the MBR to the NPR-extracted cohorts of SLE or RA using the mothers’ unique personal identification number, which is assigned to all permanent residents in Sweden. We required the two visits to occur before or on the start of pregnancy, defined as the last menstrual period date (LMP). The LMP date was determined by calculating the time between birth date and gestational age. One mother could contribute multiple pregnancies in this study. We excluded pregnancies exposed to known teratogenic medications (warfarin, phenytoin, valproate, thalidomide, antineoplastic agents, lithium, isotretinoin, misoprostol) during the first trimester or births with a diagnosis of genetic syndromes or chromosomal abnormalities during pregnancy or within 1 year of birth. The study population selection is depicted in Supplementary Figs S1 and S2, available at *Rheumatology* online. Supplementary Tables S1 and S2, also available at *Rheumatology* online, provide details on ICD codes and Anatomical Therapeutic Chemical (ATC) codes used to identify diagnoses and medications. This study was approved by the Regional Ethics Review Board in Stockholm (SLE Linkage: 2021–01148).

### Exposure

First trimester is considered the biologically plausible timeframe during which an exposure can affect the risk of MCM [29]. Therefore, the main exposure was defined as the mother having ≥1 HCQ dispensation (ATC code: P01BA02) from LMP to LMP + 90 days. Since HCQ has a long half-life (∼40 days) [30], a pregnancy could be exposed to HCQ during the first trimester if the mother used HCQ even before pregnancy. Thus, we conducted a sensitivity analysis considering a birth/pregnancy exposed if the mother received ≥1 HCQ dispensation during 3 months preceding pregnancy and the first trimester (LMP − 90 to LMP + 90). In both definitions, the unexposed group was defined with no HCQ dispensations during LMP − 90 to LMP + 90 to minimize misclassification of unexposed pregnancies. Dispensation data were collected from the Prescribed Drug Register, which includes information on all dispensations for prescribed drugs in Sweden from July 2005 [31].

Within the exposed group, average HCQ daily dose (in mg/day) was estimated by dividing the total dispensed amount during the relevant exposure window by the assumed duration of usage (90 and 180 days for the first and second exposure definitions, respectively) (Supplementary Table S1, available at *Rheumatology* online). We then categorized HCQ daily dose into two groups: ≥300 mg/day and <300 mg/day (300 mg/day is the commonly prescribed dose in Sweden).

### Outcome

The outcome was any MCM diagnosed in infants within 1 year of birth, identified from the MBR and NPR using ICD-10 codes. MCM was defined according to the European Surveillance of Congenital Anomalies (EUROCAT) and the Swedish ICD-10 codes were aligned with the 2022 EUROCAT guide 1.5 [32]. We assessed total MCM and by subgroup (Supplementary Table S2, available at *Rheumatology* online). We required ≥1 ICD code from the MBR or ≥1 ICD-coded hospitalization or ≥2 ICD-coded outpatient specialist visits of the same MCM subgroup. There is no universal consensus on detection duration for MCM, and previous studies used varying periods. Thus, for sensitivity analyses, we assessed MCM within different timeframes: 3 months of birth and 2 years of birth.

### Other variables

We collected information on potential confounders for the HCQ-MCM association. Maternal characteristics (age at conception, first-trimester BMI, first-trimester smoking, parity) were obtained from the MBR. The mothers’ country of birth, education level and income level were collected from the Longitudinal Integrated Database for Health Insurance and Labour Market Studies (LISA) [33]. A wide range of maternal comorbidities (e.g. autoimmune diseases, cancer, epilepsy, diabetes, hypertension, renal diseases) were derived from the MBR and the NPR, requiring ≥1 ICD-coded visit (≥2 visits for autoimmune diseases) any time before LMP + 90. Medication use within 6 months before pregnancy and the first trimester (e.g. corticosteroids, other DMARDs, NSAIDs) was retrieved from the Prescribed Drug Register. Disease duration (time from the date of reaching SLE or RA inclusion criteria to LMP), number of healthcare visits within 2 years before pregnancy (as a proxy for healthcare utilization), history of spontaneous abortion, and calendar year were also investigated. A full list of variables, their definitions, and data sources are described in Supplementary Table S1, available at *Rheumatology* online.

### Statistical analysis

The unit of analysis was birth or pregnancy. We described MCM subgroups and pregnancy characteristics by HCQ exposure with means and standard deviations for continuous variables and percentages for categorical variables. To control for confounding, inverse probability of treatment weighting (IPTW) based on propensity score (PS) was used. The PS is the probability of receiving HCQ during the first trimester, estimated using a logistic regression model with the potential confounders mentioned above as predictors. IPTW utilized PS to balance baseline characteristics between exposure groups by weighting each pregnancy by its inverse probability of being HCQ-exposed or unexposed. Weights were 1/PS if exposed and 1/(1 − PS) if unexposed and used to create synthetic populations. No trimming or weight stabilization was performed since there were no extreme weights. We assessed balance before and after IPTW by considering a standardized mean difference (SMD) of <10% good balance. IPTW was applied instead of PS matching since we aimed to estimate the average treatment effect in the entire population, which aligns with the current recommendation that HCQ should be used for all pregnant women with SLE unless contraindicated [2, 3, 5].

Modified Poisson regression models with robust variance estimated RRs and corresponding 95% CIs comparing HCQ-exposed with HCQ-unexposed pregnancies in the original population (unadjusted) and the weighted population (adjusted). Separate analyses were conducted for SLE and RA cohorts. Random-effects meta-analyses were used to pool the results from the two cohorts.

We performed several sensitivity analyses using different assessment windows for exposure and/or outcome as described above. Additionally, if there were >5 births with MCM in each HCQ daily dose group, we compared MCM risk between dose group *vs* unexposed. Separate PS and IPTW analyses were applied for different exposure comparisons.

Since the MBR is a birth-based register, the present analysis did not capture pregnancies ending in abortion (spontaneous or induced) that may have a higher risk of MCM. Therefore, we did a sensitivity analysis examining whether abortion risks differed by HCQ exposure. We used data from the Swedish Pregnancy Register (2013–2021) to identify SLE pregnancies. This register includes data on pregnancy and childbirth, including abortion, starting at the first antenatal care visit (week 8–12), and therefore capturing pregnancies with smaller gestational age than the MBR [34]. We did not use this register for the main analysis because it did not have full coverage of the Swedish population during the study period. This sensitivity analysis utilized a pregnancy population that overlaps with the MBR-derived main population. Those pregnancies of ≥22 weeks were already included in the main population, but those of <22 weeks were not.

Missing values were present for smoking, BMI, country of birth and socioeconomic status variables and included as a missing indicator category. All analyses were performed with R version 4.2.1 (R Foundation for Digital Computing, Vienna, Austria). Significance level was set at 5%.

## Results

### Pregnancy characteristics

The SLE cohort (*n* = 1007 pregnancies of 708 mothers) included 454 HCQ-exposed and 553 unexposed pregnancies. The RA cohort (*n* = 2500 pregnancies of 1799 mothers) comprised 144 exposed and 2356 unexposed pregnancies. In both cohorts, the mothers of exposed pregnancies *vs* unexposed had comparable age at conception (SLE: 32; RA: 33), a higher level of education (SLE: 63% *vs* 57%; RA: 68% *vs* 57%), and a lower prevalence of first-trimester smoking (SLE: 3% *vs* 5%; RA: 1% *vs* 4%) (Table 1). Overall, the exposed pregnancies appeared to have more severe disease with a greater burden of comorbidities, including pregestational hypertension, pregestational diabetes and mental disorders, as well as treatment with more medications, such as corticosteroids and DMARDs. Within the exposed pregnancies, most had an estimated HCQ daily dose of <300 mg (SLE: 74%; RA: 82%). A full description of the study cohorts is found in Supplementary Tables S3 and S4, available at *Rheumatology* online.

**Table 1.** Pregnancy characteristics by first-trimester HCQ exposure in SLE and RA cohorts, 2006–2021 in Sweden

Characteristic	SLE cohort (*n* = 1007)		RA cohort (*n* = 2500)	
	Unexposed (*n* = 553)	Exposed (*n* = 454)	Unexposed (*n* = 2356)	Exposed (*n* = 144)
Age at conception, mean (s.d.), years	31.81 (4.73)	31.38 (4.57)	32.67 (4.58)	32.78 (4.50)
Parous, *n* (%)	326 (59.0)	237 (52.2)	1382 (58.7)	79 (54.9)
Education level ≥13 years of education, *n* (%)	315 (57.0)	287 (63.2)	1350 (57.3)	98 (68.1)
Nordic country of origin, *n* (%)	469 (84.8)	370 (81.5)	2131 (90.4)	114 (79.2)
First trimester smoking, *n* (%)	28 (5.1)	15 (3.3)	92 (3.9)	<5
First trimester obesity (BMI ≥30 kg/m^2^), *n* (%)	64 (11.6)	48 (10.6)	304 (12.9)	25 (17.4)
Estimated HCQ daily dose, *n* (%)				
<300 mg/day	NA	334 (73.6)	NA	28 (19.4)
≥300 mg/day	NA	120 (26.4)	NA	116 (80.6)
Maternal comorbidity (any time before LMP + 90), *n* (%)				
Anaemia	175 (31.6)	155 (34.1)	40 (1.7)	<5
Autoimmune disease	151 (27.3)	105 (23.1)	68 (2.9)	14 (9.7)
Pregestational diabetes	15 (2.7)	17 (3.7)	69 (2.9)	6 (4.2)
Pregestational hypertension	146 (26.4)	164 (36.1)	222 (9.4)	21 (14.6)
Renal disease	31 (5.6)	17 (3.7)	11 (0.5)	<5
Mood disorder	118 (21.3)	103 (22.7)	24 (1.0)	<5
Mental disorder	16 (2.9)	16 (3.5)	17 (0.7)	<5
History of spontaneous abortion	158 (28.6)	137 (30.2)	7 (0.3)	<5
Maternal medication use (from 6 months before pregnancy until LMP + 90), *n* (%)				
Steroid	196 (35.4)	240 (52.9)	1002 (42.5)	97 (67.4)
Other DMARD/biologics	146 (26.4)	161 (35.5)	874 (37.1)	64 (44.4)
Statin	<5	6 (1.3)	6 (0.3)	<5
NSAIDs	66 (11.9)	71 (15.6)	669 (28.4)	50 (34.7)
Folic acid supplement	55 (9.9)	80 (17.6)	548 (23.3)	56 (38.9)
Psycholeptics	48 (8.7)	43 (9.5)	131 (5.6)	10 (6.9)
SSRI	39 (7.1)	55 (12.1)	105 (4.5)	9 (6.2)
Opioids	43 (7.8)	43 (9.5)	264 (11.2)	19 (13.2)
Healthcare utilization (number of visits in the previous 2 years before LMP + 90), *n* (%)				
0–5	150 (27.1)	76 (16.7)	1674 (71.1)	81 (56.2)
6–10	149 (26.9)	109 (24.0)	454 (19.3)	46 (31.9)
>10	254 (45.9)	269 (59.3)	228 (9.7)	17 (11.8)

LMP: last menstrual period; NA: not applicable; SSRI: selective serotonin reuptake inhibitor.

### Major congenital malformations

In the SLE cohort, there were 49 MCM events in 36 births (3.6%). The number of births with ≥1 MCM was 19 (3.4%) in the unexposed group and 17 (3.7%) in the exposed group. In the RA cohort, there were 168 MCM events in 110 births (4.4%) with 102 (4.3%) births in the unexposed and 8 (5.6%) in the exposed. In both cohorts, cardiac malformations were the most prevalent subgroup (51% in SLE; 42% in RA), followed by urinary tract malformations (Supplementary Tables S5 and S6, available at *Rheumatology* online). There were 8/36 (22%) births with multiple MCMs in SLE and 31/110 (28%) in RA. No clear patterns of malformation were observed.

### Association between first-trimester HCQ exposure and MCM

In the SLE cohort, balance was achieved for all characteristics (SMD < 10%), after applying IPTW (Supplementary Table S3, available at *Rheumatology* online). However, in the post-IPTW weighted RA cohort, imbalances were present for several variables, including age at conception, income, education, first-trimester smoking and BMI, pregestational diabetes, and mood disorders (Supplementary Table S4, available at *Rheumatology* online). Consequently, these variables were adjusted for in the modified Poisson models.

**Figure 1.**
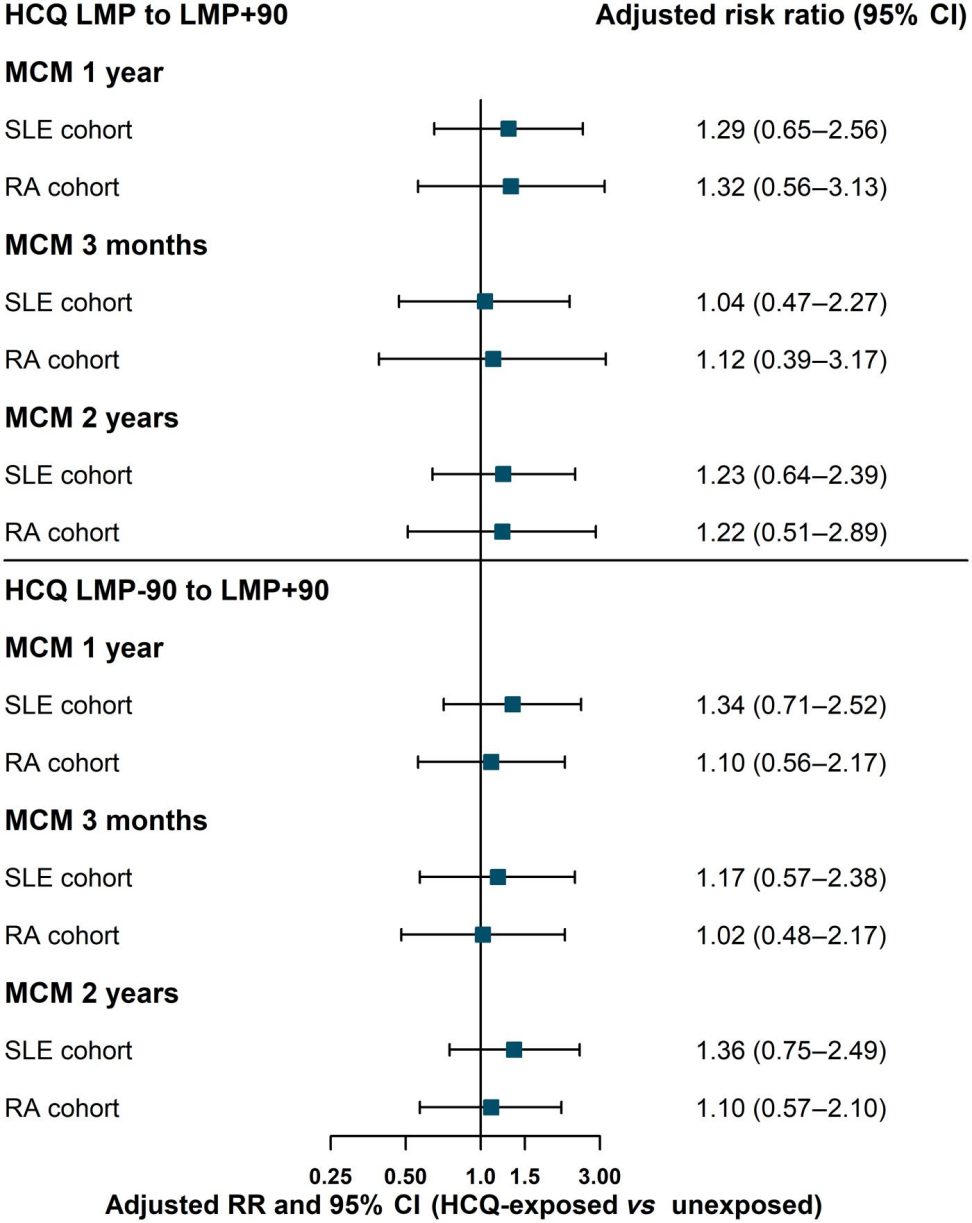
Adjusted RRs and 95% CIs of the HCQ–MCM association in SLE and RA cohorts. Main analysis: exposure window from LMP to LMP + 90. Sensitivity analysis: exposure window LMP − 90 to LMP + 90. HCQ: hydroxychloroquine; LMP: last menstrual period date; MCM: major congenital malformation; RR: risk ratio

In the SLE cohort, there was a slightly higher MCM risk in the exposed group; however, this difference was not statistically significant (adjusted RR 1.29; 95% CI: 0.65, 2.56) (Table 2, Fig. 1). The adjusted risk difference was small and not statistically significant (0.009; 95% CI: −0.016, 0.034), meaning that there were nine additional births with an MCM per 1000 births among exposed compared with unexposed. Sensitivity analyses using an extended exposure window (LMP − 90 to LMP + 90) and/or varying the outcome ascertainment period yielded similar estimates (Table 2).

**Table 2.** Association between maternal HCQ exposure and infant MCM in SLE

Exposure and outcome	MCM risk in the unexposed group, *n* (%)	MCM risk in the exposed group, *n* (%)	**Adjusted risk ratio** ^a^	95% CI
HCQ use during the first trimester as the exposure (*n* = 1007)				
MCM 1 year	19/553 (3.4)	17/454 (3.7)	1.29	0.65, 2.56
MCM 3 months	16/553 (2.9)	12/454 (2.6)	1.04	0.47, 2.27
MCM 2 years	21/553 (3.8)	18/454 (4.0)	1.23	0.64, 2.39
HCQ use during three months before pregnancy and first trimester as the exposure (*n* = 1127)				
MCM 1 year	19/553 (3.4)	24/574 (4.2)	1.34	0.71, 2.52
MCM 3 months	16/553 (2.9)	18/574 (3.1)	1.17	0.57, 2.38
MCM 2 years	21/553 (3.8)	27/574 (4.7)	1.36	0.75, 2.49

a Adjusted model refers to the models performed in the weighted sample after applying inverse probability of treatment weighting. The adjusted variables include age at conception, first trimester BMI, first trimester smoking, parity, mother’s country of birth, education level, income level, disease duration, healthcare utilization, calendar year, history of spontaneous abortion, anaemia, asthma, COPD, autoimmune diseases, cancer, epilepsy, mental disorders, mood disorders, schizophrenia, pregestational diabetes, pregestational hypertension, renal diseases, hospitalized infections, corticosteroids, DMARDs, NSAID, statins, folic acid, opioids, psycholeptics and SSRI. HCQ: hydroxychloroquine; MCM: major congenital malformation.

Similar findings were found in RA with an increased but not statistically significant risk in the exposed group *vs* unexposed (adjusted RR 1.32; 95% CI: 0.56, 3.13) (Table 3, Fig. 1). The adjusted risk difference was 0.013 (95% CI: −0.033, 0.059). Results from sensitivity analyses were also non-significant with lower RRs *vs* the main analysis (Table 3). The unadjusted RRs of all analyses are shown in Supplementary Table S7, available at *Rheumatology* online.

**Table 3.** Association between maternal HCQ exposure and infant MCM in RA

Exposure and outcome	MCM risk in the unexposed group, *n* (%)	MCM risk in the exposed group, *n* (%)	**Adjusted risk ratio** ^a^	95% CI
HCQ use during the first trimester as the exposure (*n* = 2500)				
MCM 1 year	102/2356 (4.3)	8/144 (5.6)	1.32	0.56, 3.13
MCM 3 months	80/2356 (3.4)	6/144 (4.2)	1.12	0.39, 3.17
MCM 2 years	109/2356 (4.6)	8/144 (5.6)	1.22	0.51, 2.89
HCQ use during 3 months before pregnancy and first trimester as the exposure (*n* = 2579)				
MCM 1 year	102/2356 (4.3)	12/223 (5.4)	1.10	0.56, 2.17
MCM 3 months	80/2356 (3.4)	10/223 (4.5)	1.02	0.48, 2.17
MCM 2 years	109/2356 (4.6)	13/223 (5.8)	1.10	0.57, 2.10

a Adjusted model refers to the models performed in the weighted sample after applying inverse probability of treatment weighting. The adjusted variables include age at conception, first trimester BMI, first trimester smoking, parity, mother’s country of birth, education level, income level, disease duration, healthcare utilization, calendar year, history of spontaneous abortion, anaemia, asthma, COPD, autoimmune diseases, cancer, epilepsy, mental disorders, mood disorders, schizophrenia, pregestational diabetes, pregestational hypertension, renal diseases, hospitalized infections, corticosteroids, DMARDs, NSAID, statins, folic acid, opioids, psycholeptics and SSRI. HCQ: hydroxychloroquine; MCM: major congenital malformation.

Outcome analysis for dose group was only conducted for the <300 mg/day group *vs* unexposed in the SLE cohort, and the adjusted RR indicated no association (1.09; 95% CI: 0.50, 2.37) (Supplementary Table S8, available at *Rheumatology* online). Outcome analysis was not performed for the other dose groups since there were <5 births with MCM in each dose group of the RA cohort and significant post-IPTW imbalances for the ≥300 mg/day group in the SLE cohort.

The pooled estimate showed that HCQ exposure was not statistically significantly associated with MCM risk (pooled RR 1.30; 95% CI: 0.76, 2.23). The pooled RRs from sensitivity analyses were lower and reported in Supplementary Table S7, available at *Rheumatology* online.

### Investigation of differential abortion

From the Swedish Pregnancy Register, 696 pregnancies with prevalent SLE were identified. Only six pregnancies (0.9%) were aborted. Of these, two (33%) were exposed (Supplementary Table S9, available at *Rheumatology* online).

## Discussion

In this register-based nationwide cohort study, HCQ exposure was not associated with a significantly increased risk of MCM. When considering HCQ use also during the 3 months before pregnancy and/or varying MCM assessment periods, results remained similar.

Findings from epidemiological investigations on the HCQ–MCM association are limited and inconclusive. Before 2015, most available studies examining this association reported no significantly increased risk of MCM, but they included a limited number of exposed pregnancies, making it difficult to provide a reliable effect estimate [13, 18, 35]. They also did not adequately adjust for confounding. The largest study, which included 194 exposed and 174 unexposed pregnancies, reported an RR of 3.11 (95% CI: 0.99, 9.77), but it was based on very few events (*n* = 6) [14].

Several recent studies, which were better powered and used more effective confounding adjustment methods, also did not report a significant association. A Danish nationwide cohort study of 446 births employing PS matching found no increased MCM risk associated with first-trimester HCQ use (RR 0.91; 95% CI: 0.38, 2.18) [9]. The MotherToBaby Pregnancy Study comparing MCM risk in 279 HCQ-exposed to 279 disease-matched unexposed pregnancies (OR 1.18; 95% CI: 0.61, 2.26) and to 279 healthy unexposed pregnancies (OR 0.76; 95% CI: 0.28, 2.05) did not find a significantly increased risk of malformations [36]. Other studies also revealed non-significant associations [14, 16, 37]. However, the work by Huybrechts *et al.* used claims data in the USA and applied rigorous PS analyses, resulting in a sample size of >20 000 pregnancies with a rheumatic disorder and revealing a slightly increased risk (RR 1.26; 95% CI: 1.04, 1.54) [19]. Interestingly, when stratified by dose, the group with a daily HCQ dose <400 mg did not have an increased risk (RR 0.95; 95% CI: 0.60, 1.50), and it was only the group with a daily HCQ dose ≥400 mg with a significantly increased risk (RR 1.33; 95% CI: 1.08, 1.65) [19]. They did not examine the >400 mg/day group separately, which could include pregnant women with a higher BMI as HCQ dosing could be influenced by body weight [38, 39]. High BMI has been found to be associated with an increased risk of MCM [40, 41], yet BMI was not adjusted for in the study from the USA. Also, doses exceeding 400 mg a day are not recommended according to FDA prescribing information [42] and rheumatology societies [4, 39]. Thus, it could be that those in the high dose group possessed other characteristics linked to being prescribed a high daily dose and also related to MCM risk that were not controlled for in the analysis, leading to an observed increased risk. Our dose group-stratified analysis was low-powered but still suggested a lower RR in the lower dose group. Therefore, it might be important that future research examines HCQ daily dose more thoroughly.

In comparison with previous studies on the same subject, our findings of non-significant associations are consistent with the majority, helping to expand evidence on the drug’s safety. On the other hand, the lack of significance could also stem from limited power with few MCM events. Limited power is a substantial threat to the reliability of congenital malformation research in general and particularly studies involving pregnant women with autoimmune diseases. In fact, the CIs in our study and most previous ones are relatively wide. However, it should be noted that the point estimates are around the null with a small deviation (0.8–1.3) [10, 14, 19, 36].

The variation between the current findings and others may be attributed to various factors. First, most prior studies considered pregnancies with all types of rheumatic diseases, making it challenging to draw conclusions since the disease itself may play a role in explaining the observed findings [10, 19, 14]. In contrast, our analysis focused specifically on SLE or RA, creating more specific populations and providing disease-specific estimates. Second, MCM assessment window varies between studies. While most studies, including ours, evaluated 1-year risk, the study from the USA examined MCM within 3 months of birth. This could affect the risk estimates since longer assessment duration can lead to more MCM being detected [43]. Another factor is that our study and others used different analysis methods. We employed IPTW, which estimates the average treatment effect in the entire population (i.e. the effect we would observe if all SLE or RA pregnancies were treated with HCQ), aligning with the current recommendations for SLE. Conversely, other studies applied PS matching, which estimates the average treatment effect among those pregnancies actually receiving HCQ.

The use of register data and nationwide cohorts minimizes selection bias and enhances our study’s generalizability. We employed IPTW to rigorously control for confounding by indication, with important confounders, such as smoking, BMI, treatments and comorbidities, that were not adjusted for in most prior studies. The novel application of IPTW to examine the HCQ–MCM association allowed us to estimate the average treatment effect for the entire population. Notably, our analysis focused on indication-specific populations, specifically isolating SLE and RA cohorts rather than including pregnancies with any rheumatic disease. This refined approach is beneficial for handling confounding, particularly considering the underlying disease’s role in MCM risk and the reasons for being treated with HCQ. Furthermore, we evaluated first-trimester HCQ exposure also considering pre-pregnancy use, which is pharmacokinetically relevant for HCQ, while most prior studies only assessed HCQ use within the first trimester. Although a larger sample size would have improved power, we nevertheless included a considerable number of indication-specific HCQ-exposed pregnancies compared with most prior studies, helping to expand the current body of evidence on HCQ’s safety for fetal development.

Several limitations should be acknowledged. First, aborted pregnancies with a potentially higher MCM risk were not included, hence underestimating MCM risk. If MCM-induced abortions were more commonly exposed, the RR might have been underestimated. However, our investigation on abortion in late first trimester or later using the Swedish Pregnancy Register (not restricting to livebirths) indicated that abortion was not common and it was more prevalent in the unexposed group. This would lead to overestimation of the RR. Moreover, differential abortion has been shown to not be a significant source of bias in studies looking at outcomes among livebirths [44], even though our study also included stillbirths. Second, exposure and outcome misclassification is expected to be minimal thanks to the utilization of national registers. However, drug dispensations may not imply actual use and adherence. Thus, some truly unexposed pregnancies could have been non-differentially misclassified as exposed, potentially biasing the estimates towards null. Because dispensation duration is not available in the Drug Register, we estimated daily dose based on our assumption about duration (90 days or 180 days), likely resulting in misclassification of exact daily dose. However, the categorization into < or ≥300 mg/day should be more reliable. Additionally, given the observational design, unmeasured confounding cannot be entirely ruled out as, for example, data on alcohol consumption and disease activity are lacking. However, we addressed this by adjusting for a wide range of characteristics, including alcohol use disorders, and medications. Finally, the applicability of our findings to countries other than Sweden or those with universal healthcare access is uncertain.

A substantial body of literature is available to support the benefits and safety of HCQ for pregnancies with rheumatic diseases, including reducing flare risk, disease activity, corticosteroid dose and congenital heart block [6–9, 45]. In a recent meta-analysis, HCQ was not associated with increased risks of adverse pregnancy outcomes, such as preterm birth and preeclampsia [46]. Given this context, our finding of a non-significant association between HCQ and MCM further supports the perspective that HCQ’s benefits during pregnancy outweigh the potential risks in general and the MCM risk in particular. MCM is a serious outcome and its perceived risk can substantially affect treatment for SLE or RA pregnancy. Thus, accurate findings to confirm the association are tremendously important. It is essential for healthcare providers to thoroughly assess the risk–benefit ratio of HCQ and to communicate effectively with mothers to make informed decisions, ultimately optimizing maternal and fetal health outcomes.

## Supplementary material

Supplementary material is available at *Rheumatology* online.

## Supplementary Material

keae168_Supplementary_Data

## Data Availability

It is not possible to publicly share the individual-level data used in this project due to the legal framework governing the raw data. For requests for study data, please contact the corresponding author.
