# Supplementary material for: Hydroxychloroquine in lupus or rheumatoid arthritis pregnancy and risk of major congenital malformations: a population-based cohort study
Source: Rheumatology (Oxford). 2024 Mar 13;64(1):117–25. doi: 10.1093/rheumatology/keae168 (PMC11701318; doi:10.1093/rheumatology/keae168)
Supplement: keae168_Supplementary_Data [file keae168_supplementary_data.docx]

**Hydroxychloroquine in lupus or rheumatoid arthritis pregnancy and risk of major congenital malformations: A population-based cohort study**

Ngoc V. Nguyen^1^, Elisabet Svenungsson^2^, Annica Dominicus^1^, Maria Altman^1^, Karin Hellgren^1^, Julia F. Simard^1,3^, Elizabeth V. Arkema^1^

^1^Clinical Epidemiology Division, Department of Medicine Solna, Karolinska Institutet, Stockholm, Sweden

^2^Division of Rheumatology, Department of Medicine Solna, Karolinska Institutet, Karolinska University Hospital, Stockholm, Sweden

^3^Division of Immunology and Rheumatology, Department of Medicine, Stanford School of Medicine, and Department of Epidemiology and Population Health, Stanford School of Medicine, Stanford, California, USA

**SUPPLEMENTARY MATERIALS**

[**Figure S1.** Flowchart of study population selection - Systemic lupus erythematosus cohort 2](#_Toc149313586)

[**Figure S2.** Flowchart of study population selection - Rheumatoid arthritis cohort 3](#_Toc149313587)

[**Table S1.** Variable definitions, data sources, and ICD/ATC codes for study population selection, exposure, and covariate assessment 4](#_Toc149313588)

[**Table S2.** ICD-10 codes used to assess major congenital malformations derived from EUROCAT 8](#_Toc149313589)

[**Table S3.** Characteristics of SLE pregnancy/birth cohort before and after applying IPTW in the main analysis 9](#_Toc149313590)

[**Table S4.** Characteristics of RA pregnancy/birth cohort before and after applying IPTW in the main analysis 13](#_Toc149313591)

[**Table S5.** Distribution of malformations by subgroup and exposure group in the SLE cohort (MCM event as the count unit) 17](#_Toc149313592)

[**Table S6.** Distribution of malformations by subgroup and exposure group in the RA cohort (MCM event as the count unit) 18](#_Toc149313593)

[**Table S7.** Unadjusted and adjusted risk ratios and 95% confidence intervals of the HCQ-MCM association in the SLE cohort, RA cohort, and the pooled estimates from meta-analyses 19](#_Toc149313594)

[**Table S9.** Proportions of abortion among SLE pregnancies identified from the Swedish Pregnancy Register (2013-2021) 21](#_Toc149313595)

**
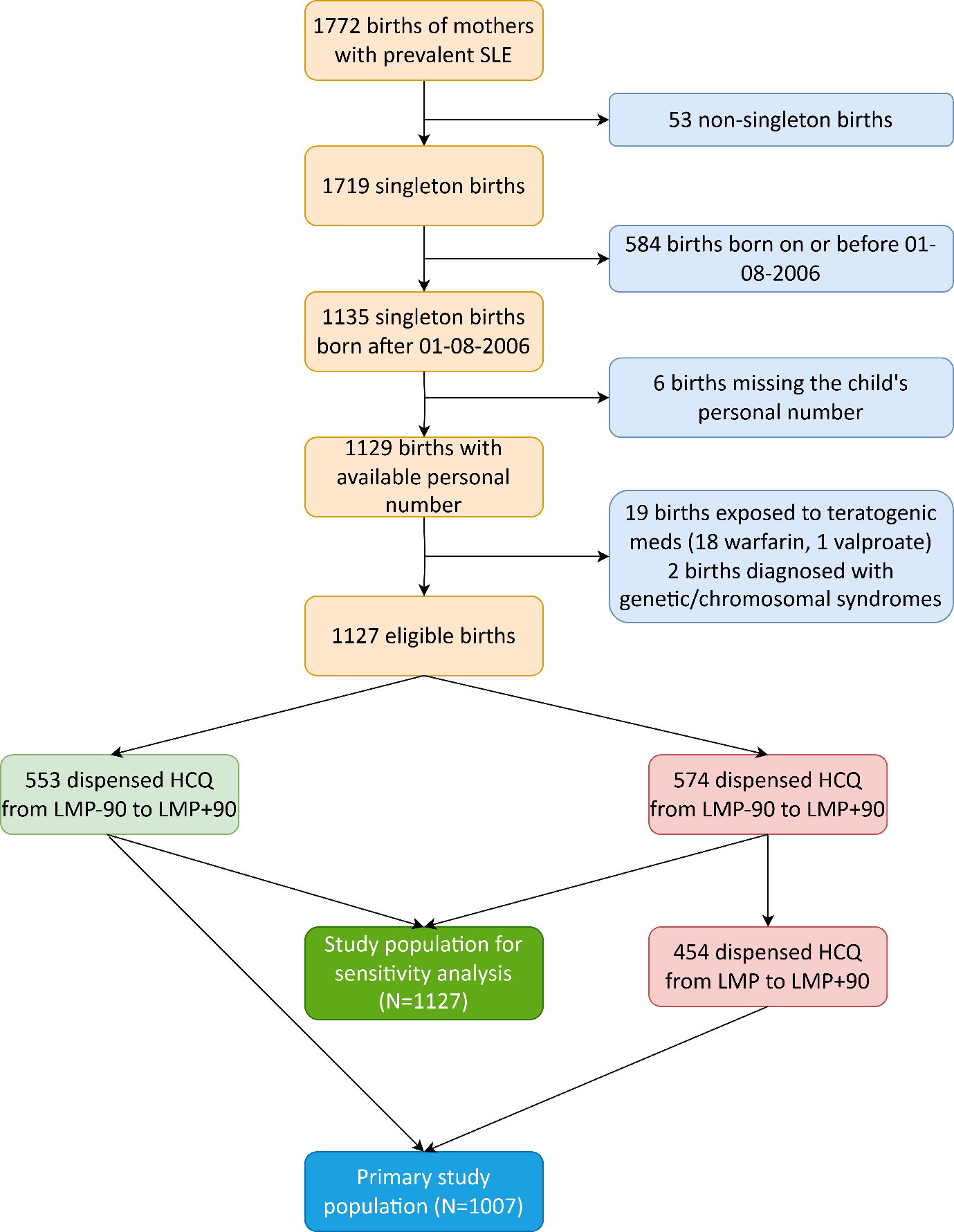
**

# **Figure S1. Flowchart of study population selection - Systemic lupus erythematosus cohort**

SLE: Systemic lupus erythematosus; HCQ: Hydroxychloroquine; LMP: Last menstrual period

**
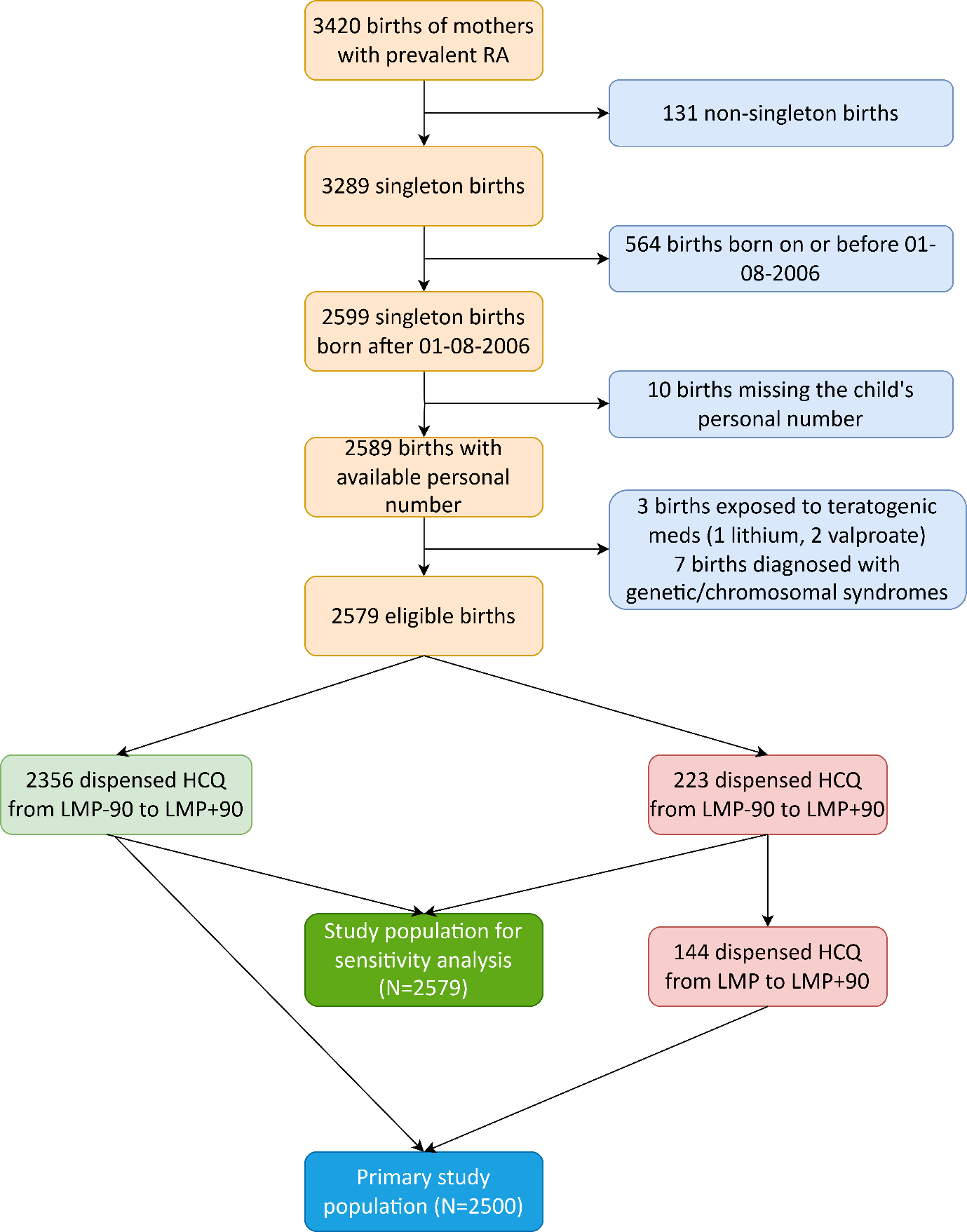
**

# **Figure S2.** Flowchart of study population selection - Rheumatoid arthritis cohort

RA: Rheumatoid arthritis; HCQ: Hydroxychloroquine; LMP: Last menstrual period

# Table S1. Variable definitions, data sources, and ICD/ATC codes for study population selection, exposure, and covariate assessment

| Variable | Definition & Data source | ICD/ATC code, categorization |
| --- | --- | --- |
| *To select study populations* | | |
| SLE | NPR: ≥2 ICD-coded visits on different dates, ≥1 visit with a specialist typically treating SLE/RA:   - SLE: rheumatology, dermatology, nephrology, internal medicine, pediatrics - RA: rheumatology, internal medicine | ICD-8: 734.1  ICD-9: 710.0  ICD-10: M32.1-M32.9 |
| RA |  | ICD-8: 712,10; 712,20; 712,38; 712,39  ICD-9: 714A-C, 714W, 719D  ICD-10: M05, M060, M062, M063, M068, M069, M123 |
| LMP | Time between birthdate and gestational age. | MBR |
| Teratogenic medications | PDR: ≥1 dispensation during LMP to LMP+90 | |
| Phenytoin |  | N03AB02, N03AB52 |
| Valproic acid |  | N03AG01 |
| Warfarin |  | B01AA03 |
| Antineoplastic agents |  | L01 |
| Thalidomide |  | L04AX02 |
| Isotretinoin |  | D10BA01 |
| Lithium |  | N05AN01 |
| Misoprostol |  | A02BB01, G02AD06 |
| Genetic syndromes, microdeletions, and chromosomal abnormalities | NPR, MBR: ≥1 ICD-coded visit from LMP to 1 year after birth | ICD-10: Q86; P350; P351; P354; P358; P371; D821; Q611; Q612; Q613; Q751; Q754; Q77; Q780; Q781; Q782; Q783; Q784; Q785; Q786; Q787; Q788; Q796; Q800; Q824; Q850; Q851; Q87; Q90; Q91; Q92; Q93; Q96; Q97; Q98; Q99 |
| *Exposure:* HCQ | PDR | P01BA02 |
| HCQ daily dose calculation* | PDR | In our data, HCQ is dispensed per 100-tablet package with a strength of 200 mg each table.  Calculation: HCQ daily dose in mg =  (Total number of packages (variable ANTAL) from all dispensations within the assessment period x 100 tablets x 200 mg)/Assumed duration of treatment in days (90 days for use within the first trimester or 180 days for use within three months before pregnancy until the end of the first trimester). |
| *Covariates* |  |  |
| Age at conception | Age at LMP date: MBR | Continuous variable in years  Age group: <20, 20–24, 25–29, 30–34, 35–39, ≥40. |
| Parity | MBR | Nulliparous/Multiparous |
| Country of birth | LISA | Nordic/ Non-Nordic |
| Education level in the year before the LMP year | LISA | ≤9 years, 10-12 years, ≥13 years |
| Income level in the year before the LMP year | LISA | <100, 100-299, 300-599, ≥600 (x1000 SEK) |
| 1^st^ trimester smoking | MBR | Yes/No |
| 1^st^ trimester BMI | MBR | Continuous variable in kg/m^2^  Group: Underweight (<18.5 kg/m^2^)  Normal weight (18.5–24.9 kg/m^2^)  Overweight (25.0–29.9 kg/m^2^)  Obese (≥30.0 kg/m^2^) |
| Disease duration | NPR, MBR: Duration from date of reaching SLE/RA criteria to LMP | Continuous variable in years |
| Calendar year | MBR: Year of LMP | 2006-2010; 2011-2016; 2017-2021 |
| Healthcare utilization | NPR: Number of inpatient and outpatient visits within 2 years before LMP+90 | 0-5; 6-10; >10 |
| *Maternal comorbidities* | | |
| Asthma | ≥1 ICD-coded visit any time before LMP+90 | ICD-8 & ICD-9: 493  ICD-10: J45–J46 |
| Anemia |  | ICD-8 & ICD-9: 280-289  ICD-10: D50-D80 |
| Cancer |  | ICD-10: C00-C97  ICD-9: 140-209 |
| COPD |  | ICD-10: J41; J42; J43; J44.1; J44.8; J44.9  ICD-9: 491A; 491B; 491X; 491; 492; 496  ICD-8: 490; 491,01; 491,02; 491,04; 491,04; 492 |
| Epilepsy |  | ICD-10: G40, G41  ICD-9: 345  ICD-8: 345 |
| Renal disease |  | Prior renal disease  NPR: ≥ 1 ICD code any time before LMP  N00-N06, N08.2, N08.5, N16.2, N16.4, N16.8, N18, N19, N26, Z94.0, Operation codes for kidney biopsy KAB00, KAB01 580-584  Y29,01 580-587  V42A |
| Mood disorders |  | ICD-10: F30-F39  ICD-9: 296  ICD-8: 296 |
| Mental disorder |  | ICD-10: F10-F19  ICD-9: 303-305  ICD-8: 303-305 |
| Schizophrenia |  | ICD-10: F20-F29  ICD-9: 295  ICD-8: 295 |
| Autoimmune disease | ≥2 ICD-coded visits any time before LMP+90 | ICD-10: E063; E270; M30; M31; M45; K900; E10; G610; E050; K50; K51; G35; M33; M321; M328; M329; M350; M34  ICD-9: 245C; 255E; 446; 720A; 579A; 250; 357A; 242A; 555; 556; 340; 710D; 710E; 710A; 710C; 710B  ICD-8: 245,03; 255,10; 446; 712,40; 726,99; 250; 269,10; 357; 242,00; 563,00; 563,10; 340; 569,02; 716; 734,10; 734,90; 734,0 |
| Pregestational diabetes | NPR: ≥1 ICD code  PDR: ≥1 anti-diabetic medication, excluding insulin MBR: ≥1 ICD code | ICD-10: E11; O240-O243  ICD-9: 250; 648A  ICD-8: 250  ATC: A10 (excluding A10A) |
| Pregestational hypertension | NPR: ≥1 ICD code  PDR: ≥1 antihypertensive medication  MBR: ≥1 ICD code | ICD-10: I10-I15; O10; O11  ICD-9: 642C; 642H; 401-405  ICD-8: 400-404  ATC codes: C02AB; C02CA; C07; C08; C09 |
| Serious infection | ≥1 ICD-coded visit in the inpatient register as the primary discharge diagnosis during LMP to LMP+90 | A00–B99; D73.3; E06.0; E32.1; G00–G07; H00.0; H44.0; H60.0; H60.1; H60.2; H60.3; H66; H67; H70; I30.1; I40.0; J00–J22; J32; J34.0; J36; J38.3; J39.0; J39.1; J44.0; J85; J86; K04.4; K04.6; K04.7; K10.2; K11.3; K12.2; K14.0; K57.0; K57.2; K57.4; K57.8; K61; K63.0; K65.0; K65.9; L00–L08; L30.3; M00; M01; M46.2; M46.3; M46.4; M60.0; M64.5; M65.0; M71.0; M71.1; M72.6; M86; N10–N12; N13.6; N15.1; N15.9; N30.0; N30.8; N34.0; N39.0; N41.2; N43.1; N45; N48.2; N61; N70–N74; N75.1; O23; O26.4; O41.1; O75.3; O85; O86; O88.3; O91; O98 |
| History of spontaneous abortion | MBR: variable TIDSPOAB  NPR: ≥1 ICD-coded visit any time before LMP | ICD-10: O021; O03  ICD-9: 632-634  ICD-8: 643-644 |
| *Maternal medication use* | PDR: ≥1 dispensation during 6 months before pregnancy until the end of 1^st^ trimester | |
| Systemic corticosteroids |  | H02AB |
| DMARDs | Cyclophosphamide, sulfasalazin, methotrexate, leflunomide, cyclosporin, azathioprine, natriumaurotiomalat, auranofin, chloroquine, tacrolimus, sirolimus, mycophenolate | L01BA01; L04AA13; L04AD01; L04AX01; L04AX03; M01CB01; M01CB03; P01BA01; L01AA01; D11AH01; L04AD02; L04AA10; S01XA23; L04AA06 |
| Biologics | Rituximab, abatacept, etanercept, infliximab, adalimumab, certolizumabpegol, golimumab, anakinra, tocilizumab, tofacitinib, baricitinib, sarilumab, belimumab | L01XC02; L04AA24; L04AA26; L04AA29; L04AA37; L04AB01; L04AB02; L04AB04; L04AB05; L04AB06; L04AC03; L04AC07; L04AC14; L04AA26 |
| Statin |  | C10AA |
| NSAIDs |  | M01A |
| Folic acid supplement |  | B03BB01 |
| Psycholeptics |  | N05 |
| SSRI |  | N06AB |
| Opioid |  | N02A |

ICD: International classification of disease; ATC: Anatomical therapeutic chemical classification; MBR: Medical birth register; NPR: National patient register; PDR: Prescribed drug register; LISA: Swedish Longitudinal Integrated Database for Health Insurance and Labour Market Studies; SLE: Systemic lupus erythematosus; RA: Rheumatoid arthritis; HCQ: Hydroxychloroquine; LMP: Last menstrual period; SD: Standard deviation; COPD: Chronic obstructive pulmonary disease; BMI: Body mass index; DMARD: Disease-modifying anti-rheumatic drug; NSAID: Non-steroidal anti-inflammatory drug; SSRI: Selective serotonin reuptake inhibitor

# **Table S2.** ICD-10 codes used to assess major congenital malformations derived from EUROCAT

| **Organ system** | **ICD-10 codes** |
| --- | --- |
| Nervous system | Q00-Q07 |
| Eye | Q10-Q15  Except Q101-Q103; Q105; Q135 |
| Ear, face, and neck | Q16-Q18  Except Q170-Q175; Q179; Q180-Q182; Q184-Q187; Q189 |
| Congenital heart defects | Q20; Q21; Q22; Q23; Q24; Q25; Q26  Except Q246; Q261; Q250 and preterm; Q256 and preterm |
| Respiratory system | Q300; Q32; Q33; Q34  Except Q320; Q322; Q331; Q336 |
| Orofacial clefts | Q35; Q36; Q37  Except Q357 |
| Digestive system | Q38; Q39; Q40; Q41; Q42; Q43; Q44; Q45; Q790  Except Q381; Q382; Q400; Q401; Q430; Q444 |
| Abdominal wall defects | Q792; Q793; Q795 |
| Urinary system | Q60; Q61; Q62; Q63; Q64; Q794  Except Q610; Q627; Q633 |
| Genital organs | Q50; Q51; Q52; Q54; Q55; Q56  Except Q501; Q502; Q505; Q523; Q525; Q527; Q544 |
| Limb | Q65; Q660; Q679; Q68; Q69; Q70; Q71; Q72; Q73; Q74  Except Q653; Q654; Q655; Q656; Q658; Q659; Q680; Q683; Q684; Q685 |
| Other | Q750; Q893; Q894; Q044; Q411; Q412; Q418; Q710; Q712; Q720; Q722; Q730; Q793; Q206; Q240; Q890; Q893 |

# **Table S3.** Characteristics of SLE pregnancy/birth cohort before and after applying IPTW in the main analysis

| **Characteristics** | **Before IPTW (original sample)** | | | **After IPTW (weighted sample)** | | |
| --- | --- | --- | --- | --- | --- | --- |
|  | **HCQ use during the first trimester as the exposure** | | | | | |
|  | **No (N=553)** | **Yes (N=454)** | **SMD** | **No (N=1011)** | **Yes (N=1001)** | **SMD** |
| Maternal age, mean (SD) | 31.81 (4.73) | 31.38 (4.57) | 0.092 | 31.64 (4.78) | 31.56 (4.59) | 0.017 |
| Parous (%) | 326 (59.0) | 237 (52.2) | 0.136 | 565.3 (55.9) | 561.6 (56.1) | 0.003 |
| Income level (x1000 SEK), n (%) |  |  | 0.108 |  |  | 0.045 |
| <100 | 272 (49.2) | 243 (53.5) |  | 526.0 (52.0) | 525.0 (52.4) |  |
| 100-299 | 189 (34.2) | 146 (32.2) |  | 329.2 (32.6) | 324.7 (32.4) |  |
| 300-599 | 86 (15.6) | 62 (13.7) |  | 147.4 (14.6) | 145.2 (14.5) |  |
| ≥600 | 5 (0.9) | 3 (0.7) |  | 7.1 (0.7) | 6.5 (0.7) |  |
| Missing | 1 (0.2) | 0 (0.0) |  | 1.0 (0.1) | 0.0 (0.0) |  |
| First trimester smoking, n (%) |  |  | 0.094 |  |  | 0.028 |
| No | 499 (90.2) | 414 (91.2) |  | 918.5 (90.9) | 908.0 (90.7) |  |
| Yes | 28 (5.1) | 15 (3.3) |  | 42.5 (4.2) | 47.2 (4.7) |  |
| Missing | 26 (4.7) | 25 (5.5) |  | 49.7 (4.9) | 46.2 (4.6) |  |
| First trimester BMI group, n (%) |  |  | 0.077 |  |  | 0.064 |
| Underweight | 11 (2.0) | 14 (3.1) |  | 22.9 (2.3) | 29.5 (2.9) |  |
| Normal weight | 311 (56.2) | 258 (56.8) |  | 573.5 (56.7) | 555.6 (55.5) |  |
| Overweight | 126 (22.8) | 102 (22.5) |  | 234.7 (23.2) | 249.9 (25.0) |  |
| Obese | 64 (11.6) | 48 (10.6) |  | 105.3 (10.4) | 97.8 (9.8) |  |
| Missing | 41 (7.4) | 32 (7.0) |  | 74.4 (7.4) | 68.7 (6.9) |  |
| Country of origin, n (%) |  |  | 0.089 |  |  | 0.034 |
| Non-Nordic | 7 (1.3) | 7 (1.5) |  | 14.3 (1.4) | 15.0 (1.5) |  |
| Nordic | 77 (13.9) | 77 (17.0) |  | 145.7 (14.4) | 156.0 (15.6) |  |
| Missing | 469 (84.8) | 370 (81.5) |  | 850.7 (84.2) | 830.4 (82.9) |  |
| Education level, n (%) |  |  | 0.161 |  |  | 0.023 |
| ≤9 years | 44 (8.0) | 30 (6.6) |  | 78.0 (7.7) | 71.9 (7.2) |  |
| 10-12 years | 191 (34.5) | 131 (28.9) |  | 324.1 (32.1) | 328.0 (32.8) |  |
| ≥13 years | 315 (57.0) | 287 (63.2) |  | 600.4 (59.4) | 593.5 (59.3) |  |
| Missing | 3 (0.5) | 6 (1.3) |  | 8.2 (0.8) | 8.1 (0.8) |  |
| SLE duration (years), mean (SD)  (from date of reaching SLE criteria for inclusion to LMP date) | 7.09 (4.90) | 6.77 (4.99) | 0.064 | 7.01 (4.99) | 7.00 (4.88) | 0.002 |
| Calendar year, n (%) |  |  | 0.485 |  |  | 0.018 |
| 2006-2010 | 200 (36.2) | 78 (17.2) |  | 279.5 (27.7) | 281.5 (28.1) |  |
| 2011-2016 | 217 (39.2) | 188 (41.4) |  | 394.6 (39.0) | 395.1 (39.4) |  |
| 2017-2021 | 136 (24.6) | 188 (41.4) |  | 336.7 (33.3) | 324.9 (32.4) |  |
| **Maternal comorbidity any time before LMP+90 (except for first trimester hospitalized infection), n (%)** | | | | | | |
| Asthma | 41 (7.4) | 35 (7.7) | 0.011 | 74.7 (7.4) | 69.8 (7.0) | 0.016 |
| Anemia | 175 (31.6) | 155 (34.1) | 0.053 | 332.9 (32.9) | 325.0 (32.5) | 0.010 |
| Antiphospholipid syndrome | 28 (5.1) | 57 (12.6) | 0.267 | 86.6 (8.6) | 82.9 (8.3) | 0.011 |
| Autoimmune disease | 151 (27.3) | 105 (23.1) | 0.096 | 209.2 (20.7) | 200.7 (20.0) | 0.016 |
| Cancer | 9 (1.6) | 11 (2.4) | 0.056 | 20.4 (2.0) | 18.8 (1.9) | 0.011 |
| COPD | 7 (1.3) | 6 (1.3) | 0.005 | 11.6 (1.1) | 10.9 (1.1) | 0.006 |
| Epilepsy | 19 (3.4) | 18 (4.0) | 0.028 | 41.0 (4.1) | 45.2 (4.5) | 0.023 |
| Pregestational diabetes | 15 (2.7) | 17 (3.7) | 0.058 | 18.9 (1.9) | 25.7 (2.6) | 0.047 |
| Pregestational hypertension | 146 (26.4) | 164 (36.1) | 0.211 | 333.9 (33.0) | 320.7 (32.0) | 0.021 |
| First trimester hospitalized infection | 3 (0.5) | 1 (0.2) | 0.052 | 5.9 (0.6) | 2.0 (0.2) | 0.061 |
| Renal disease | 31 (5.6) | 17 (3.7) | 0.088 | 141.0 (14.0) | 133.8 (13.4) | 0.017 |
| Mood disorder | 118 (21.3) | 103 (22.7) | 0.033 | 227.6 (22.5) | 240.2 (24.0) | 0.035 |
| Mental disorder | 16 (2.9) | 16 (3.5) | 0.036 | 29.3 (2.9) | 28.8 (2.9) | 0.001 |
| Schizophrenia | 0 (0.0) | 3 (0.7) | 0.115 | 86.6 (8.6) | 82.9 (8.3) | 0.011 |
| History of spontaneous abortion | 158 (28.6) | 137 (30.2) | 0.035 | 290.8 (28.8) | 285.8 (28.5) | 0.005 |
| **Maternal medication use (from 6 months before pregnancy until LMP+90), n (%)** | | | | | | |
| Steroid | 196 (35.4) | 240 (52.9) | 0.356 | 445.1 (44.0) | 434.1 (43.3) | 0.014 |
| Other DMARD | 146 (26.4) | 161 (35.5) | 0.197 | 310.0 (30.7) | 313.6 (31.3) | 0.014 |
| Statin | 2 (0.4) | 6 (1.3) | 0.105 | 7.0 (0.7) | 8.2 (0.8) | 0.014 |
| NSAIDs | 66 (11.9) | 71 (15.6) | 0.108 | 150.0 (14.8) | 152.8 (15.3) | 0.012 |
| Folic acid supplement | 55 (9.9) | 80 (17.6) | 0.224 | 135.4 (13.4) | 143.1 (14.3) | 0.026 |
| Psycholeptics | 48 (8.7) | 43 (9.5) | 0.028 | 95.2 (9.4) | 96.1 (9.6) | 0.006 |
| SSRI | 39 (7.1) | 55 (12.1) | 0.173 | 92.3 (9.1) | 91.4 (9.1) | <0.001 |
| Opioid | 43 (7.8) | 43 (9.5) | 0.060 | 87.4 (8.6) | 86.8 (8.7) | 0.001 |
| Healthcare utilization (total number of visits in the previous 2 years before LMP+90), n (%) |  |  | 0.297 |  |  | 0.025 |
| 0-5 | 150 (27.1) | 76 (16.7) |  | 222.3 (22.0) | 230.4 (23.0) |  |
| 6-10 | 149 (26.9) | 109 (24.0) |  | 253.7 (25.1) | 245.1 (24.5) |  |
| >10 | 254 (45.9) | 269 (59.3) |  | 534.7 (52.9) | 525.9 (52.5) |  |

SLE: Systemic lupus erythematosus; HCQ: Hydroxychloroquine; IPTW: Inverse probability of treatment weighting; SMD: Standardized mean difference; LMP: Last menstrual period; SD: Standard deviation; COPD: Chronic obstructive pulmonary disease; BMI: Body mass index; DMARD: Disease-modifying anti-rheumatic drug; NSAID: Non-steroidal anti-inflammatory drug; SSRI: Selective serotonin reuptake inhibitor

# **Table S4.** Characteristics of RA pregnancy/birth cohort before and after applying IPTW in the main analysis

| **Characteristics** | **Before IPTW (original sample)** | | | **After IPTW (weighted sample)** | | |
| --- | --- | --- | --- | --- | --- | --- |
|  | **HCQ use during the first trimester as the exposure** | | | | | |
|  | **No (N=2356)** | **Yes (N=144)** | **SMD** | **No (N=2359.9)** | **Yes (N=133.9)** | **SMD** |
| Maternal age, mean (SD) | 32.67 (4.58) | 32.78 (4.50) | 0.026 | 32.66 (4.59) | 33.25 (4.30) | 0.133 |
| Parous, n (%) | 1382 (58.7) | 79 (54.9) | 0.077 | 1380.6 (58.5) | 74.1 (55.4) | 0.063 |
| Income level (x1000 SEK), n (%) |  |  | 0.485 |  |  | 0.122 |
| <100 | 1414 (60.0) | 84 (58.3) |  | 1414.6 (59.9) | 75.7 (56.6) |  |
| 100-299 | 663 (28.1) | 27 (18.8) |  | 649.7 (27.5) | 37.3 (27.9) |  |
| 300-599 | 167 (7.1) | 6 (4.2) |  | 163.2 (6.9) | 13.6 (10.1) |  |
| ≥600 | 9 (0.4) | 1 (0.7) |  | 9.4 (0.4) | 0.3 (0.2) |  |
| Missing | 103 (4.4) | 26 (18.1) |  | 123.1 (5.2) | 6.9 (5.2) |  |
| First trimester smoking, n (%) |  |  | 0.217 |  |  | 0.241 |
| No | 2142 (90.9) | 136 (94.4) |  | 2151.0 (91.1) | 123.6 (92.3) |  |
| Yes | 92 (3.9) | 1 (0.7) |  | 87.6 (3.7) | 0.6 (0.5) |  |
| Missing | 122 (5.2) | 7 (4.9) |  | 121.3 (5.1) | 9.7 (7.2) |  |
| First trimester BMI group, n (%) |  |  | 0.125 |  |  | 0.232 |
| Underweight | 52 (2.2) | 3 (2.1) |  | 51.9 (2.2) | 3.1 (2.3) |  |
| Normal weight | 1284 (54.5) | 74 (51.4) |  | 1280.8 (54.3) | 76.0 (56.8) |  |
| Overweight | 576 (24.4) | 34 (23.6) |  | 574.3 (24.3) | 27.7 (20.7) |  |
| Obese | 304 (12.9) | 25 (17.4) |  | 314.0 (13.3) | 12.4 (9.2) |  |
| Missing | 140 (5.9) | 8 (5.6) |  | 138.8 (5.9) | 14.8 (11.0) |  |
| Country of origin, n (%) |  |  | 0.318 |  |  | 0.008 |
| Non-Nordic | 225 (9.6) | 30 (20.8) |  | 241.7 (10.2) | 14.1 (10.5) |  |
| Nordic | 2131 (90.4) | 114 (79.2) |  | 2118.2 (89.8) | 119.8 (89.5) |  |
| Education level, n (%) |  |  | 0.306 |  |  | 0.247 |
| ≤9 years | 163 (6.9) | 6 (4.2) |  | 159.0 (6.7) | 2.9 (2.2) |  |
| 10-12 years | 827 (35.1) | 36 (25.0) |  | 817.6 (34.6) | 55.1 (41.2) |  |
| ≥13 years | 1350 (57.3) | 98 (68.1) |  | 1362.7 (57.7) | 75.1 (56.1) |  |
| Missing | 16 (0.7) | 4 (2.8) |  | 20.5 (0.9) | 0.7 (0.6) |  |
| RA duration (years), mean (SD)  (from date of reaching RA criteria for inclusion to LMP date) | 5.38 (3.86) | 4.37 (3.64) | 0.270 | 5.31 (3.85) | 5.10 (3.88) | 0.055 |
| Calendar year, n (%) |  |  | 0.536 |  |  | 0.243 |
| 2006-2010 | 730 (31.0) | 16 (11.1) |  | 702.3 (29.8) | 26.6 (19.8) |  |
| 2011-2016 | 1011 (42.9) | 67 (46.5) |  | 1019.8 (43.2) | 70.4 (52.6) |  |
| 2017-2021 | 615 (26.1) | 61 (42.4) |  | 637.8 (27.0) | 36.9 (27.6) |  |
| **Maternal comorbidity any time before LMP+90 (except for first trimester serious infection), n (%)** | | | | | | |
| Asthma | 27 (1.1) | 1 (0.7) | 0.047 | 26.4 (1.1) | 1.2 (0.9) | 0.024 |
| Anemia | 40 (1.7) | 3 (2.1) | 0.028 | 40.3 (1.7) | 1.1 (0.8) | 0.083 |
| Autoimmune disease | 68 (2.9) | 14 (9.7) | 0.284 | 79.3 (3.4) | 3.4 (2.6) | 0.046 |
| Cancer | 5 (0.2) | 0 (0.0) | 0.065 | 4.7 (0.2) | 0.0 (0.0) | 0.063 |
| COPD | 1 (0.0) | 0 (0.0) | 0.029 | 0.9 (0.0) | 0.0 (0.0) | 0.028 |
| Epilepsy | 2 (0.1) | 0 (0.0) | 0.041 | 1.9 (0.1) | 0.0 (0.0) | 0.040 |
| Pregestational diabetes | 69 (2.9) | 6 (4.2) | 0.067 | 71.1 (3.0) | 7.5 (5.6) | 0.127 |
| Pregestational hypertension | 222 (9.4) | 21 (14.6) | 0.159 | 228.5 (9.7) | 12.7 (9.5) | 0.007 |
| First trimester hospitalized infection | 3 (0.1) | 0 (0.0) | 0.050 | 2.8 (0.1) | 0.0 (0.0) | 0.049 |
| Renal disease | 11 (0.5) | 1 (0.7) | 0.030 | 11.3 (0.5) | 0.4 (0.3) | 0.035 |
| Mood disorder | 24 (1.0) | 0 (0.0) | 0.143 | 22.6 (1.0) | 0.0 (0.0) | 0.139 |
| Mental disorder | 17 (0.7) | 2 (1.4) | 0.065 | 21.3 (0.9) | 0.6 (0.5) | 0.051 |
| History of spontaneous abortion | 7 (0.3) | 0 (0.0) | 0.077 | 6.6 (0.3) | 0.0 (0.0) | 0.075 |
| **Maternal medication use (from 6 months before pregnancy until LMP+90), n (%)** | | | | | | |
| Corticosteroid | 1002 (42.5) | 97 (67.4) | 0.515 | 1040.1 (44.1) | 59.4 (44.4) | 0.006 |
| Conventional synthetic DMARDs | 245 (10.4) | 33 (22.9) | 0.341 | 266.0 (11.3) | 18.6 (13.9) | 0.078 |
| Biologics | 702 (29.8) | 34 (23.6) | 0.140 | 694.1 (29.4) | 51.9 (38.7) | 0.198 |
| Statin | 6 (0.3) | 0 (0.0) | 0.071 | 6.2 (0.3) | 0.0 (0.0) | 0.073 |
| NSAIDs | 669 (28.4) | 50 (34.7) | 0.136 | 678.8 (28.8) | 38.2 (28.6) | 0.005 |
| Folic acid supplement | 548 (23.3) | 56 (38.9) | 0.343 | 573.4 (24.3) | 35.7 (26.7) | 0.054 |
| Psycholeptics | 131 (5.6) | 10 (6.9) | 0.057 | 133.8 (5.7) | 5.3 (3.9) | 0.081 |
| SSRI | 105 (4.5) | 9 (6.2) | 0.080 | 107.0 (4.5) | 3.3 (2.5) | 0.112 |
| Opioid | 264 (11.2) | 19 (13.2) | 0.061 | 270.4 (11.5) | 16.0 (12.0) | 0.016 |
| Healthcare utilization (total number of visits in the previous 2 years before LMP+90), n (%) |  |  | 0.323 |  |  | 0.070 |
| 0-5 | 1674 (71.1) | 81 (56.2) |  | 1652.2 (70.0) | 94.4 (70.5) |  |
| 6-10 | 454 (19.3) | 46 (31.9) |  | 474.7 (20.1) | 24.1 (18.0) |  |
| >10 | 228 (9.7) | 17 (11.8) |  | 233.0 (9.9) | 15.4 (11.5) |  |

RA: Rheumatoid arthritis; HCQ: Hydroxychloroquine; IPTW: Inverse probability of treatment weighting; SMD: Standardized mean difference; LMP: Last menstrual period; SD: Standard deviation; COPD: Chronic obstructive pulmonary disease; BMI: Body mass index; DMARD: Disease-modifying anti-rheumatic drug; NSAID: Non-steroidal anti-inflammatory drug; SSRI: Selective serotonin reuptake inhibitor

# **Table S5.** Distribution of malformations by subgroup and exposure group in the SLE cohort (MCM event as the count unit)

| **Malformation subgroup, n (%)** | **HCQ-unexposed* (N=24)** | **HCQ-exposed* (N=25)** | **Total MCM (N=49)** |
| --- | --- | --- | --- |
| Congenital heart defects | 14 (58.3) | 11 (44.0) | 25 (51.0) |
| Urinary system | 6 (25.0) | 2 (8.0) | 8 (16.3) |
| Limb | 2 (8.3) | 6 (24.0) | 8 (16.3) |
| Orofacial clefts | 0 (0) | 3 (12.0) | 3 (6.1) |
| Genital organs | 1 (4.2) | 2 (8.0) | 3 (6.1) |
| Digestive system | 1 (4.2) | 0 (0) | 1 (2.0) |
| Eye | 0 (0) | 1 (4.0) | 1 (2.0) |

SLE: Systemic lupus erythematosus; HCQ: Hydroxychloroquine; MCM: Major congenital malformation

*: HCQ use during the first trimester as the exposure (main analysis)

# **Table S6.** Distribution of malformations by subgroup and exposure group in the RA cohort (MCM event as the count unit)

| **Malformation subgroup, n (%)** | **HCQ-unexposed* (N=160)** | **HCQ-exposed* (N=8)** | **Total MCM (N=168)** |
| --- | --- | --- | --- |
| Congenital heart defects | 66 (41.3) | 4 (50.0) | 70 (41.7) |
| Limb | 28 (17.5) | 3 (37.5) | 31 (18.5) |
| Urinary system | 12 (7.5) | 0 (0) | 12 (7.1) |
| Eye | 11 (6.9) | 1 (12.5) | 12 (7.1) |
| Orofacial clefts | 12 (7.5) | 0 (0) | 12 (7.1) |
| Genital organs | 12 (7.5) | 0 (0) | 12 (7.1) |
| Digestive system | 10 (6.3) | 0 (0) | 10 (6.0) |
| Nervous system | 4 (2.5) | 0 (0) | 4 (2.4) |
| Ear, face, and neck | 2 (1.3) | 0 (0) | 2 (1.2) |
| Abdominal wall | 1 (0.6) | 0 (0) | 1 (0.6) |
| Other | 1 (0.6) | 0 (0) | 1 (0.6) |
| Respiratory system | 1 (0.6) | 0 (0) | 1 (0.6) |

RA: Rheumatoid arthritis; HCQ: Hydroxychloroquine; MCM: Major congenital malformation

*: HCQ use during the first trimester as the exposure (main analysis)

# **Table S7.** Unadjusted and adjusted risk ratios and 95% confidence intervals of the HCQ-MCM association in the SLE cohort, RA cohort, and the pooled estimates from meta-analyses

| **Analysis** | **Unadjusted RR (95%CI)** | **Adjusted RR (95%CI)** |
| --- | --- | --- |
| HCQ LMP to LMP+90 & MCM 1 year |  |  |
| SLE cohort | 1.09 (0.57-2.07) | 1.29 (0.65-2.56) |
| RA cohort | 1.28 (0.64-2.58) | 1.32 (0.56-3.13) |
| Pooled | NA* | 1.30 (0.76-2.23) |
| HCQ LMP to LMP+90 & MCM 3 months |  |  |
| SLE cohort | 0.91 (0.44-1.91) | 1.04 (0.47-2.27) |
| RA cohort | 1.23 (0.54-2.76) | 1.12 (0.39-3.17) |
| Pooled | NA* | 1.06 (0.57-1.99) |
| HCQ LMP to LMP+90 & MCM 2 years |  |  |
| SLE cohort | 1.04 (0.56-1.94) | 1.23 (0.64-2.39) |
| RA cohort | 1.20 (0.60-2.41) | 1.22 (0.51-2.89) |
| Pooled | NA* | 1.23 (0.73-2.08) |
| HCQ LMP-90 to LMP+90 & MCM 1 year |  |  |
| SLE cohort | 1.22 (0.67-2.20) | 1.34 (0.71-2.52) |
| RA cohort | 1.24 (0.69-2.22) | 1.10 (0.56-2.17) |
| Pooled | NA* | 1.22 (0.77-1.94) |
| HCQ LMP-90 to LMP+90 & MCM 3 months |  |  |
| SLE cohort | 1.08 (0.56-2.10) | 1.17 (0.57-2.38) |
| RA cohort | 1.32 (0.69-2.51) | 1.02 (0.48-2.17) |
| Pooled | NA* | 1.10 (0.65-1.84) |
| HCQ LMP-90 to LMP+90 & MCM 2 years |  |  |
| SLE cohort | 1.24 (0.71-2.16) | 1.36 (0.75-2.49) |
| RA cohort | 1.26 (0.72-2.20) | 1.10 (0.57-2.10) |
| Pooled | NA* | 1.23 (0.79-1.92) |

*We only performed the pooled analysis using adjusted estimates from the individual cohorts

**Table S8.** Association between maternal HCQ exposure during the first trimester with a daily dose of <300 mg/day and risk of MCM in the infants in the SLE cohort

| **Exposure and outcome** | **MCM risk in the unexposed group, n (%)** | **MCM risk in the exposed group, n (%)** | **Adjusted risk ratio*** | **95%CI** |
| --- | --- | --- | --- | --- |
| **HCQ use during the first trimester as the exposure (N=887)** | | | | |
| MCM 1 year | 19/553 (3.4) | 11/334 (3.3) | 1.09 | 0.50-2.37 |
| MCM 3 months | 16/553 (2.9) | 7/334 (2.1) | 0.70 | 0.28-1.72 |
| MCM 2 years | 21/553 (3.8) | 12/334 (3.6) | 1.05 | 0.50-2.22 |
| **HCQ use during three months before pregnancy and first trimester as the exposure (N=1,067)** | | | | |
| MCM 1 year | 19/553 (3.4) | 22/514 (4.3) | 1.30 | 0.68-2.49 |
| MCM 3 months | 16/553 (2.9) | 16/514 (3.1) | 1.09 | 0.52-2.27 |
| MCM 2 years | 21/553 (3.8) | 25/514 (4.9) | 1.34 | 0.73-2.48 |

# **Table S9.** Proportions of abortion among SLE pregnancies identified from the Swedish Pregnancy Register (2013-2021)

|  | **HCQ-unexposed* (N=345)** | **HCQ-exposed* (N=351)** | **Overall (N=696)** |
| --- | --- | --- | --- |
| **Pregnancy loss/termination, n (%)** |  |  |  |
| No | 341 (98.8) | 349 (99.4) | 690 (99.1) |
| Yes | 4 (1.2) | 2 (0.6) | 6 (0.9) |

SLE: Systemic lupus erythematosus; HCQ: Hydroxychloroquine

*: HCQ use during the 1^st^ trimester as the exposure (main analysis)
